# State Medicine*Read before the meeting of the Medical Editors of the United States, at New Orleans.

**Published:** 1885-07

**Authors:** Frank S. Billings

**Affiliations:** Pathologist to the New York Polyclinic School of Medicine


### THE JOURNAL

OF

### COMPARATIVE MEDICINE AND SURGERY.

VOL. VI. JULY, 1885. No. 3.

### ORIGINAL COMMUNICATIONS.

### Art. XVI.—STATE MEDICINE.*

BY FRANK S. BILLINGS,
Pathologist to the New York Polyclinic School of Medicine.

There is probably no branch of the medical profession for which all questions with reference to State Medicine have more vital interest, than the veterinarian. While the medical practitioner is assured of his social position so long as he attends to his practice, it is essential to the veterinarian that his value as an agent upon the vital matters of public health, as well as upon all questions with regard to contagious animal diseases, be recognized by the State. This is not the case in the United States, and least of all in Massachusetts, hence it is that I have thought it well that we should have some ideas of what is understood by "State Medicine."

The careful student of the medical institutions of the United States must most certainly admit that there is much to be desired and much rottenness to be rooted out before they can become, not only what they should be, but worthy of so wealthy a country and so energetic and practical a people.

* Read before the meeting of the Medical Editors of the United States, at New Orleans.

In speaking of the "medical institutions" of the country, you must not think that the schools alone are to be the subject for consideration. *They* form but a unit among many, but unfortunately for many schools, as well as for the people and the nation, it is the most important and fundamental unit in the whole organism. If the foundation is weak, the whole superstructure must be in a like condition. Before entering upon the discussion of this, the educational part of our question, it is essential that we come to a clear understanding of what we mean by the "medical institutions" of a country. To this end it is necessary that we define our ideas of medical science.

The ultimate endeavor, or purpose, of medical science, as we understand it to-day, is not to educate practitioners in medicine, i. e., to make doctors alone, but rather to subject all disease to the most searching intellectual, physical, chemical, macro and microscopical analysis possible, in the hope that we may thus be enabled to discover, not only the nature of a given disease, but also the nature of its causes—for all diseases have their internal organismal, or predisposing causes, as well as their external or exciting causes—in order that we may elucidate means by which to prevent the generation of such disease so far as possible in the future. Up to the present time our endeavors in this direction have been almost entirely limited to diseases of an infectious or contagious nature. Our work in the future must also include the so-called sporadic, or, as I would term them, common diseases of life, the chief cause of which is to be sought in the utter ignorance of humanity of the laws of life. The bacillus of ignorance plays a much greater role in the causation of disease than that of phthisis, cholera, or septicæmia, and will be much more difficult to combat and expose.

The world wants an applied physiology suitable to the intelligence of the educated and thinking masses as well as it does for the medical student.

While prevention is the alpha and omega of our endeavors, it is not always of possible attainment, so that the world demands the practitioner to treat its diseases as well as the investigator to search into their nature and causes, and to make experiments as to their treatment.

The individual man naturally looks upon the practitioner as the more important of the two, because it is with him alone that he comes in immediate contact. For the masses, however, and by that we mean the nation as a whole, the investigator is by far the more important, though ignorance still so rules the world that none but the exceptionally educated have as yet recognized the value of this fact. The practitioner needs to learn that even he would be at an intellectual standstill were it not for the men in the laboratories whom he so often styles the "theoretical and unpractical men of his profession."

The medical institutions of a country are:

First, the schools; second, the boards of health, or its preventive force; third, the practitioners.

The science of medicine is the great life-saving service of the world.

The medical institutions of this country should be as much a part of its "life-saving service" as that brave body of men that have been so successfully organized along our sea-board and lake coasts during the past ten years.

Every intelligent person must admit that if the medical institutions of this country should be part of its life-saving service (and in reality almost the whole of it), that they should also be organized and regulated in the same manner as any other branch of the public service. The division of this service has already been stated, but the classification has also another practical value.

We have divided this service thus: I. The educational part. II. The entire hygienic system. These two branches must be entirely regulated and supported by the State. III. The practical division which must be controlled by State, as it is subject to the Hygienic Department, but receives its remuneration directly from the person benefited.

The medical schools are the foundation of this service. It has already been said "that if the foundation is weak the entire organization must be of the same nature."

To the schools we have to look as the nurseries out of which we are to draw our supplies for all parts of this extended and complicated branch of the public service.

What, then, is the work of the schools?

1. The education of scientists capable of teaching others so that they may follow in their paths and become able and enthusiastic investigators into the nature of disease, and everything having an etiological connection therewith.

2. The education of practitioners in the principles which have resulted from such scientific researches, as well as the practical experiences of the world.

Do our schools fulfill these conditions? Emphatically, No!

If we make a careful survey of medical literature, we shall be made aware of the fact that the medical schools of the United States are rendered conspicuous by the almost total absence of contributions to original research.

Practitioners are, and have been, well educated at some of these schools, but the fundamentals upon which teaching depends are nearly all borrowed from continental authors; the practical side is largely American, and if due regard is given to what our European confréres are doing, leaves nothing to be desired in that direction.

Why is it that our schools do not fulfill all the demands we have a right to make upon them? I would have the answer impressed very deeply upon the minds of those having anything to do with veterinary education in this country.

Because there is not a single medical school in the United States that is properly supported and regulated by the State; because there is not a medical school in this country where all the teachers have been selected for their abilities not only to teach well but to investigate; because there are no schools offering free opportunities for men of ability to go and work and lecture as private docents, thereby giving genius a chance for self-support and enthusiasm an opportunity to extend over the land; because we have no school where education is free, where the one purpose is to fulfill its whole duty as a branch of the public service. These ends can only be attained by a national institute of medical science—including animal and vegetable physiology and pathology—supported by the government and regulated by a well selected national board of health. The time has come when we should begin to make Washington the central light of American civilization.

Endowed schools can never meet these requirements. All such schools are private, in that they form no part of the public service. They can never shake off the evil influences which they have inherited from their inception. Speculative schools will not even let science enter their doors, only as they must, in the form of honeyed misrepresentations, to attract the students flying round after cheap and easily gotten diplomas. Up to the present time, every medical school that has been established in the United States has been for the purpose—only of making doctors. The majority of those that I have classed as "endowed" are honest schools, and were established in the earlier days of their respective States, to meet a public necessity. The country was young then; almost its entire population being busied in developing it. Drones and sick people were a luxury the earlier settlers could not afford to have with them. The first they drove out, and they desired the sickness driven out of the others. They wanted doctors. The intelligence of the country has not augmented in any proportion to its increase in wealth and population. It still fails to grasp the mission of medical science in the world, or to realize that its executive institutions should be a part of the public service of the State. It does not realize that research is the foundation of all knowledge, or that "knowledge is the power" by which only we are enabled to prevent the ravages of disease.

It still demands curers only. If we are called before a committee of one of our legislative bodies with regard to any disease that is making itself unpleasantly frequent, they ask us, "what can you do to cure it," not what is its nature. If we speak of making researches into that and after its causes, and the necessity of time and money to make investigations with the hope of preventing it, they immediately put us down as a "crank."

The other class of schools, the speculative, to which may be added those that are run upon the "subscription system"—although incorporated, are only to be condemned. They are the result of unscrupulous greed on the part of selfish and ambitious men who desire to increase their influence and incomes—at any cost to their profession—by being thus enabled to pose as "the learned professor" before an unreflecting public, as well as from the fees of students for whom they feel no responsibility either with regard to the quantity or quality of education given them; and on the other hand, of the action of ignorant, irresponsible and unthinking legislators that are willing to charter anything. In their endeavors to get students, the faculty of these speculative schools have very capacious maws—they send the most astonishing catalogues broadcast over the land, in which they state their advantages one over the other. These illustrated pamphlets remind one very strongly of the advertisements of summer hotels and boarding houses. The buildings and surroundings are very imposing—upon paper—and then there is very great similarity in the characters of the proprietors. In the one case, they care nothing about the intellectual food which their patrons receive; as in the other, with regard to the bodily—they are both alike in demanding prompt and full payment. In 1876 we had some sixty, while in 1885 we are credited with having 134 medical schools in these United States. In some States there are as many as eight of these institutions, and in at least one of our cities there is the same number. The majority of these schools are of the speculative variety. As they are in no sense of the word, of service to the public, there is no valid reason for their existence.

No branch of the public service of a State should have even the suspicion of speculation hovering over it, though all parts of this service should be conducted after the most rigid and exact business principles.

What do we mean by "business principles" with reference to medical schools? The returns which the people have a right to expect are the same here as in any other branch of the governmental service; they are: The devotion to duty of the public official at any cost to himself. Only the enthusiast is fit to officiate in this branch of the service. This spirit can never result from our present school system.

It is said "that there are no classes in America." This is not true, for we have every class of worthy and unworthy society; but the most important one of all to the continued development of a country and the preservation of the public health is the scientific class. These are the sappers and miners who prepare the way and make possible the success of every class in the world. Without this class, of what worth is, the accumulated riches of the world when disease threatens?

In the face of cholera the proud merchant for once bows in proper humility to knowledge, the practitioner for the nonce becomes modest, and the world looks only at the work of the investigators. The people cease to cry for "cure alls," but rather demand that the disease be prevented from landing on our shores.

Let us look at some of the evil results of our medical school system.

1st. Not being scientific, i. e., investigating schools, they have not exerted any influence for the advancement of medical science in the country.

(*a*) The reason for this is, 1st. That they are not State schools; and 2d, Lack of funds. This condition has been rendered still worse by the idiotic chartering of speculative, opposition, or special schools of medicine in numerous States and cities, so that the incomes from students' fees of the original and endowed school has been rendered unnecessarily small.

(*b*) The endowed schools again have been too frequently obliged to depend upon men as teachers in the fundamental branches of medical education that had no especial fitness for the work, except that they were born rich and that some relative had been one of the original founders.

(*c*) These two conditions have exerted a most unfortunate influence in limiting the freedom of the trustees in their selection of teachers, and preventing them from seeking genius wherever they could find it.

2d. The great number of these medical schools, and the fact that the majority of them are speculative, tends to lowering the standard of education, and to the production of an utter want of uniformity in the quantity and quality of education acquired by graduates from different schools either in the same or different States, or what is still more disgraceful, in the same city.

3d. This evil condition of things has resulted in bringing unnecessary disgrace upon an honorable profession, and of lowering the appreciation of the same in the public mind. More incompetent than really trustworthy graduates have thus been enabled to represent themselves as "M.Ds." or "doctors."

4th. So little appreciation have the people of the majority of the States of the value of a thorough education in medicine, that it has thus far been impossible to find intelligence enough in their representatives to regulate the practice of medicine in order to protect the public and reward honest endeavor by passing a law to prevent charlatans from using the title "doctor" as a means of deception.

Medicine being a part of the public service, it should be regulated by the State. If one introduces the questions herein brought to discussion to the attention of the most intelligent of our teachers, or practitioners, he is invariably met with one answer, viz.: "Yes, these things are all too true, but how are they to be obviated?—you must remember that we are but a young country yet."

I may be mistaken, or perhaps over-conceited, but I think I have given more thoroughly honest and sceptical study to these questions than any man in the country, for the reason that I desired to have all these evils avoided with regard to veterinary schools. The causes of these unfortunate conditions are not to be sought in the youthfulness of the United States in comparison with other nations—for it must be remembered that the settlers and immigrants to this country brought and bring with them the average intelligence of their native lands—but rather to two other circumstances, and nothing else.

1st. Our English inheritances; 2d. That we have a representative form of government, i. e., are a republic, which does not represent the acme of intelligence in the country by any means. There is, or has been, no better appreciation of the true relation of medical science to the public in Britain than in America. There is no such thing as organized State medicine either in Britain or any of her dependencies. There, as here, all the medical schools are either endowed or chartered speculative institutions. There, as here, you can find the only veterinary schools in the world that are conducted on the "subscription plan" regardless of every principle of professional honor. They are all "doctor" makers, nothing more. The government assumes no responsibility as to how the instruction is being given, or the examinations conducted at the British medical schools. There is scarcely any original research at these schools. The British government is opposed to it—by enacting a law against the intelligent use of vivisection.

The British government is an improvement upon ours in some respects, in that, if it does not regulate the medical and veterinary schools, it has allowed the respective professions to select their own examining and diploma-granting bodies, and by prohibiting to charlatanry the use of the titles belonging to the regular graduate, has shown that it has some regard for honesty and its duty to the public. Britain has neither a well regulated and organized public health or veterinary police service.

All this ignorance our ancestors brought with them, and we have not improved an iota since that day; in fact we are far behind Britain yet, for she does not cherish, protect and nourish the pestiferous diploma-mills which disgrace many of the States of this Union.

In the United States, the people of which boast so much about their prosperity and intelligence, there is scarcely any such thing as State medicine, no efficient boards of health, no veterinary police organization, no organized market and meat inspection, no trustworthy milk inspection, no inspection of dairies to guarantee to the public that the milk they consume and feed their babes upon is not directly from poorly fed and poorly housed cows, or from animals diseased with garget, or what is worse, tuberculosis.

Not a State in this Union gathers any reliable statistics upon all these questions. Our State boards of health are more or less a farce; composed of men the majority of whom have no knowledge of their duties. Not one of them has a suitable laboratory at its disposal wherein investigations and experiments can be constantly made.

The question is, Why are these conditions peculiarly characteristic of English-speaking countries? It is an axiomatic fact that the more English a people the more inefficient their government upon all questions of the kind we are considering. Another fact of like character has long ago made itself evident to every careful student of the inductive sciences, namely, that original research has prospered, and at present flourishes, among the great civilized nations of the world in direct ratio to the non-republican character of their governments.

In other words, the history of republicanism gives direct evidence that thus far it has been utterly unequal to the appreciation of the great cardinal principle of national prosperity that "public health is public wealth;" that this condition cannot be attained or maintained without the careful regulation of the medical institutions of the country as a part of the public service. That the entire system is embraced under the term of State medicine. From this practical point of view that "public health is public wealth," every republic and every extremely liberal government has been and is an abject failure. Again, whenever we see science flourishing and original research supported under a republican banner, as in France, it is not due to republic statesmanship, but rather that the republic has inherited and has had sense enough, or else was indolent enough, to retain the good of the monarchical institutions which in other ways it superseded. Monarchical France made possible the work of Bichat, Cruvelheir, Laennec, Duypeyton, Magendie, Claude Bernard, Pasteur and others, and monarchical Germany has given opportunity to her Mueller, Helmholz, DuBois—Raymond, Webber, Rokitansky (of Austria), Ludwig, Gegenbaur, Virchow, Koch, and so many others that the world can never pay the debt it owes such governments for the facts which research has given to it, and for the advancement and improvement of the human race, by recognizing that the world owes such men a living.

Put Britain and America upon the stand, and question the hand that records the immortal deeds of the men of science. How much have you done in this direction? Remember we are speaking of State medicine only! Everything that is of public importance is of a political nature, and not one deserves a higher, more disinterested, or non-partisan statesmanship than the questions of public health. All questions regarding the medical institutions of any country, bear so intimately upon its prosperity that the professionals who neglect them or who would leave them to the considerations of the ordinary lay politicians, are false to their duties.

The question then left for us to consider is: Why is it that original research has prospered and public health organizations found the best development under energetic monarchical governments, and not under republican forms? The answer is: In governments "of the people, by the people, for the people," the masses never think of anything outside of their daily routine life; they follow in the ruts, and leave the interests of the country entirely in the hands of party leaders who make a business of politics and not a science of statesmanship.

So long as the cholera is in Europe and business prosperous in this country, the American citizen scarcely thinks it of any account. With it at our doors, threatening his own life and that of those dear to him, he suddenly wakes up and begins to wonder why he has not been better informed in regard to the matter. Then he wants legislation.

There is one practical result to monarchical governments that is to be observed where all the medical institutions are regulated by the State. It is this: Whatever tends to render the State prosperous as a whole, is directly beneficial to the government, and whatever tends to injure any great national interest, and the public health is the greatest, is at once felt by the government. National prosperity means success to the government; national adversity means anxiety, distress, and, perhaps, destruction to the ruling family. Let us make this apparent by some examples:

The loss of 100,000 men from some disease, say cholera, to either France or Germany (the other remaining exempt through the energy and activity of its public health service) would be liable to have an instantaneous effect upon the peace of Europe and the stability of some of its governments. While the "Rinderpest" is but a cattle disease, a severe eruption of it in either of the two deciding nations of Europe, could cause such losses both in food, material and means, as to sensibly weaken it, and hence strengthen indirectly, its opponent. Therefore, an active veterinary police as well as a public health system, is in the interests of every individual citizen, consequently it is the duty of every intelligent government to see that such organizations exist, self-interest has also made them obligatory to continental governments. Semi-barbarian Russia can show us the way in these directions in her most civilized portions.

In speaking of these questions previously, I mentioned the comparatively isolated and favorable situation of our country politically. We are nominally, as well as virtually, the monarch of all we survey. We have no equally powerful neighbors which make it a matter of careful necessity that our government makes a constant and careful study of every little thing which may advance or interfere with the resources of the country. Public health is not, as in Germany, a national necessity, for we are not in the condition in which Heine placed the German citizen when he said that "they married in order to supply food for French cannon." There is as much truth as satire in those words for all the nations of Europe, and if cholera, or any other pest anticipates the wants of the cannon, and the food supply is reaped by the unsparing harvester on his own account, or if this same harvester carries devastation among the herds and flocks of the nation, thereby taking away the means of feeding and owning the cannon's food, they acquire it at all the less cost to the opposing nation. Not one of these questions strike directly at either the prosperity or vitality of our government, and although I did not think of it before, the isolated situation of Britain is also of importance to her, the many wars she has waged having had but little direct bearing on these questions.

But if public health is not a public necessity from this existence or prosperity point of view, to a republic in our favorable position, it is still of vast importance to us, a people from another stand point, and that is the public health. The more healthful a nation is the more it prospers, the more energetic its people, is a question that needs no proof.

A fierce epidemic that threatened the lives of the laborers in the mining regions of Pennsylvania, could, if long continued, almost stagnate the business of this country. The rinderpest starting on the Atlantic seaboard, and following the track of the noted equine distemper of '72, might impoverish the country to a great degree and cause incalculable suffering. The cholera may cost us some valuable and many uncounted lives, and much misery this next summer. But not one or all of these things will, or can, cause the least trouble to the government of the United States. Its geographical position has rendered the nation non-sensitive to any such things.

I think I have made my meaning clear, that it becomes a matter of vital necessity for all governments to support original research in all sciences, and especially in medicine, and that though our government is not endangered, it is no less its duty to fulfil every obligation to the people, to study the needs of the country and not wait until absolute necessity awakens the people, so that they demand this or that.

All true governments must be parental whatever their constitutional form may be. The more truly parental, the more it treats the people, in every part of it, with the impartiality of true parents, the more it studies to ward off injurious influences from all parts, and to strengthen all by chemical researches as to the value of their ore products, by physical ones as to their influence for this or that interest, by researches at national laboratories for new uses of all the products of the country, and last and above all, the more it studies the public health in every particular, the greater will be the public wealth of the nation.

Coming back, then, to the question of our medical institution, we have seen that we are in a really bad condition to fulfill these demands. The first difficulty is to know what to do with our school system and its irregularities in the directions indicated. We are all aware that there are altogether too many cheap and irresponsible schools. We cannot fall back upon two examining and diploma granting bodies, the "Colleges of Physicians and Surgeons," as has been the good fortune of our brethren in Britain.

It must be made possible by concerted effort to make every school of this country begin and end its course on the same dayof the year, and have exactly the same number of courses. It must be made possible to have the education approximately the same at all of them. It must be made possible to put a stop to the chartering, or endowing, of any more schools in any State where one school exists.

We must all of us seek to strengthen the strongest schools in our respective States regardless of our alma-mater. No matter how conservative, dead, or mismanaged it may be we must seek to reform it, and never lend our hand to establish an opposition school. We must remember that medicine is not a business, but a profession, and the schools must be made to pay in delivering the best goods possible.

As to a system of examination for all the schools, one hardly knows what to say. Given State schools, then none but the teachers are suitable for examiners. Under our system, it would seem as if the examining power should rest in the State board of health. A board of health should be composed of a number of the most advanced specialists upon hygiene in the States, one or more hygienic engineers and an accomplished jurist. What party they belong to, or what church they attend, makes no difference so long as they do their duty, the regulation of the practice of medicine.

Though it is now too late to make all the schools State institutions, still they should all be made to wheel into line and become a part of the public service of the State. This is not the difficult task it appears to be. The purpose of this is, that no person shall practice medicine, representing him, or herself, to be either an " M. D."—" doctor," " doctress," " surgeon " or " physician," within the boundaries of a given State, without first having past the legal examination of the State board of health and having received a license to practice, which must be recorded and then entered upon the records of the selectmen, or when possible, of the local board of health of the place where such person settles for practice. Said local authorities should at once verify the license of said practitioner by communication with the State board of health.

Those that have practiced medicine under the above conditions in a given State, should, on leaving said State, have their license endorsed stating their standing, reasons for and date of leaving. If these conditions are accepted as equal to those demanded by the board of health of another State into which said practitioner moves with intent to practice his or her profession, it should only be necessary to present said license from the original State, which, being verified, should be recorded in the new State, and the bearer thereof permitted to practice as previously mentioned.

The medical service of the State should form one comprehensive public health system; this means the regulation of the practice of medicine, the duties of boards of health, meat, market, dairy, milk inspection, etc., it includes the regular veterinary and irregular practitioner in its grasp. There should be a national board of health at Washington, and in each State a State board of health, and in every city and large town a local board of health. In the country districts, each county should have its chief public health official, and should be divided into districts, each of which should also have its officials. Not one of these should be appointed by any local authorities; all should be paid by the State; all should hold position during active life unless removed for incompetency or unfaithfulness; all should be "medical examiners." If injured during public service in any way so as to unfit him for future work, they should be subject to pension.

The medical officers of the State board must, of necessity, be at first appointed by the governor, the engineer and jurists to be selected by the medical officials and confirmed by the governor. All local officials to receive their appointment by public competition before the State board. All vacancies to be filled by the advancement of local officers that have distinguished themselves by faithfulness to their duties. The vacancies of the State board to be filled eventually from the best among the county officials. Before receiving such appointments, or better, before being eligible to competition for vacancies, every applicant should be obliged to attend a special course of study, for which the State should supply the opportunity and material free. Every State should supply its board of health with a chemical, biological and pathological laboratory where continued investigations of an examining. observational and experimental character should be conducted. Where, anyone desiring it, could also go and devote himself to special work in either of these directions, the State supplying all material free. At the same time it should equally serve the place of an advanced school in medical research under the auspices of the State, and for the instruction of candidates for the health organization. I will now close by stating how I would regulate the practice of medicine.

The title being guaranteed only to qualified persons, the hygienic organization formed, the public being left free to choose whom they please to attend their ills, the whole practicing fraternity, be it on the human or animal organism, should be obliged by law to report to the nearest State medical official the existence of the suspicion of any infectious or contagious disease in any individual of either species to whom their services should be called. The State board of health should publish a list of such diseases, as well as the laws for the proper regulation of each according to its peculiar nature.

Any violation of the above regulation should be punished with fine or imprisonment, or both, according to the nature of the case, which should be fixed by law and not left to the courts to decide. Ignorance should never be accepted as a mitigating circumstance.

